# Comprehensive Review of Genetic Association Studies and Meta-Analysis on polymorphisms in microRNAs and Urological Neoplasms Risk

**DOI:** 10.1038/s41598-018-21749-4

**Published:** 2018-02-28

**Authors:** Ligang Zhang, Meng Zhang, Hao Wang, Yangyang Wang, Jun Zhou, Zongyao Hao, Li Zhang, Chaozhao Liang

**Affiliations:** 1 Department of Urology, the First Affiliated Hospital of Anhui Medical University, Hefei, 230022 China; 2 Institute of Urology, Anhui Medical University, Hefei, 230022 China; 3 Graduate School of Anhui Medical University, Hefei, 230022 China

## Abstract

Gene expression is negatively regulated by microRNAs (miRNAs), which commonly act as tumor oncogenes or suppressors. Previous results were inconsistent concerning the relationship between polymorphisms in miRNAs and risk of urological neoplasms. Here, we conducted a comprehensive literature research on diverse databases aiming at enrolling all eligible studies up to August 31, 2016. A total of 13 publications comprising 29 case-control studies were enrolled for three polymorphisms in three miRNAs. Overall analyses suggested significant associations between miR-146a rs2910164 polymorphism and urological neoplasms risk in allelic, homozygote and recessive models. In the stratified analysis by ethnicity, we uncovered a significant association between rs2910164 polymorphism and risk of urological neoplasms in Asian populations in allelic, homozygote and recessive models. Highlighted, when stratified analysis was conducted by cancer type, rs2910164 polymorphism was also significantly associated with an increased risk of bladder cancer in allelic, homozygote and recessive models. Although for rs11614913 and rs3746444 polymorphisms, overall analyses suggested negative results, for rs11614913 polymorphism, when subgroup analysis was conducted by cancer type, a significantly decreased risk of renal cell cancer was identified in recessive model. In brief, current work indicated that miR-146a rs2910164 polymorphism is a risk factor for urological neoplasms, particularly for bladder cancer.

## Introduction

Considering statistics of global cancers, the morbidity and mortality rates of urological neoplasms rank significantly in populations. The etiology and pathogenesis of urological neoplasms have a lot in common. In order to prevent urological neoplasms, it is necessary to investigate the relevant predisposing factors, such as environmental factors, diet, intake of non-steroidal and anti-inflammatory drugs, and endogenous factors, which may affect individual risk of urological neoplasms. In addition, a new mechanism which may regulate transcription by microRNA (miRNA) has been found to be related to various diseases, including cancer^1^.

MiRNAs are a kind of non-coding single chain with 22 nucleotides in length, existing extensively in viruses, plants and animals, which are evolutionarily conserved. MiRNAs have three levels of structure: primary structure, secondary structure, tertiary structure^2^. Among them, the secondary structure of miRNA corresponds to the shape(or topology) induced by all base pairings A-U, G-C, and G-U from the single-stranded molecule. It is thus composed by matched regions, called stems (or helices), and unpaired regions, called loops. A secondary structure is essentially defined by its shape, and more precisely by the configuration of stems and the nature of unpaired bases. Such a shape can be involved in: (1) formation of the tertiary structure; (2) biological functions of the miRNA; (3) interactions with other RNAs or with proteins. Additionally, miRNAs indeed represent a crucial mechanism transcriptionally regulating gene expression. To date, plenty of evidences have indicated that mature miRNAs participated in the degradation or translational suppression of mRNA by linking to the 3′ untranslated region of target genes, and eventually related to the regulation of various critical biological activities, including cell metabolism, proliferation, differentiation, proliferation, and apoptosis, even working as tumor suppressors or oncogenes for the sake of participating in tumorigenesis through posting expression regulation of homologous target genes^3–5^. Some key miRNAs, which have been regarded as biomarkers, could ameliorate diagnosis and prediction of prognosis and treatment response for cancer patients^6^.

Single nucleotide polymorphisms (SNPs) are defined as a kind of genetic polymorphisms related to disease susceptibility, population diversity, drug metabolism and genome evolution^7–9^. It has been proposed that SNPs found in miRNA genes affect miRNA transcription, processing, and interactions with target mRNAs. SNPs in miRNA genes are widely-accepted to affect function in one of three ways: firstly, through the transcription of the primary transcript; secondly, through pri-miRNA and pre-miRNA processing; and also through effects on miRNA-mRNA interactions^10^. For example, miR-196a gene encodes many mature products including miR-196a and miR-196a*, and it also comprises rs11614913 polymorphism. In a study conducted by Hoffman *et al*.^11^, they demonstrated that relative to empty vector control, expression of miR-196a can be increased when transfected with pre-miR-196a-C or miR-196a-T in breast cancer cells. In addition, miR-196a was doubly more expressed in cells transfected with pre-miR-196a-C when compared with miR-196a-T. Namely, rs11614913 polymorphism can influence pre-miRNA processing into mature and functional form, and subsequently activating tumorigenesis. In recent years, plenty of investigators have studied the relationship between genetic polymorphisms in precursor or mature miRNA sequence and diverse cancer risks, including gastric cancer (GC)^12,13^, colorectal cancer (CRC)^14^, liver cancer^15^, gallbladder cancer (GBC)^16^, esophagus cancer (EC)^17^, bladder cancer (BCa)^18^ and prostate cancer (PCa)^19^. However, these results were controversial and inconsistent. In current work, we performed an update meta-analysis at the aim of precisely verifying the relationship between genetic polymorphisms in miRNAs and urological neoplasms risk.

## Materials and Methods

### Search strategy and study selection

We carried out a literature research for all eligible articles that investigated the relationship of three polymorphisms in miRNAs (miR-146a rs2910164; miR-499 rs3746444; miR-196a2 rs11614913) with urological neoplasms on Embase, PubMed, Science Direct, and Web of Science (up to August 31, 2016) through using the following terms: (miRNA 146a/499/196a2) AND (polymorphism OR variation) AND (carcinoma OR cancer OR neoplasm OR adenocarcinoma OR tumor OR tumour). All the fields of the retrieved articles were screened through titles and abstracts. We also checked reference lists of the review articles and enrolled articles.

Eligible studies were included while they met the following inclusion criteria: (1) assessment of microRNA 146a/499/196a2 polymorphisms and risk of urological neoplasms; (2) case-control design independently for human, and; (3) providing useful data of genotype frequencies. At the same time, the exclusion criteria were presented as follows: 1) duplicate data, 2) reports of clinical cases, comments, series, reviews and editorial and 3) insufficient data. Studies published in some other languages instead of English were also excluded. Articles involved with two or more case-control tests were regarded as two or more different studies.

### Data extraction

We reviewed carefully for each publication, and extracted the following data which conformed to the selection criteria: first author, year of publication, original country, ethnicity of the population studied, genotyping method, source of controls, cancer type, numbers for cases and controls of all genotypes, whether verified Hardy-Weinberg equilibrium (HWE). If original data of genotype frequency was not provided in relevant studies, we could send a request to the corresponding author for additional information.

### Statistical analysis

We used crude ORs and 95% CIs to evaluate the relationship of the miRNA polymorphisms and urological neoplasms under five genetic models, including allelic (B vs. A), recessive (BB vs. BA + AA), dominant (BA + BB vs. AA), homozygous (BB vs. AA), and heterozygous (BA vs. AA) models^20^ (A: wild allele; B: mutated allele). In addition, subgroup analyses were conducted by source of controls, genotyping methods, ethnicity, cancer type and HWE status. Chi-square-based Q-tests were used to check heterogeneity across all the included studies, and *P* < 0.05 level was considered statistically significant^21^. If there existed heterogeneity in the included studies, the random-effect model (DerSimonian and Laird method) was performed to assess the pooled OR, otherwise the fixed-effect models (DerSimonian and Laird method) were adopted^22^. We determined publication bias using Begg’s funnel plot and Egger’s test. Sensitivity analyses were conducted in order to assess the data stability. We removed each study involved in this meta-analysis one at a time, and the residual studies were analyzed. STATA version 12.0 was applied in carrying out all data analyses. Statistical significance was set at the level of *P* < 0.05.

## Results

### Study Characteristics

**Figure 1:**
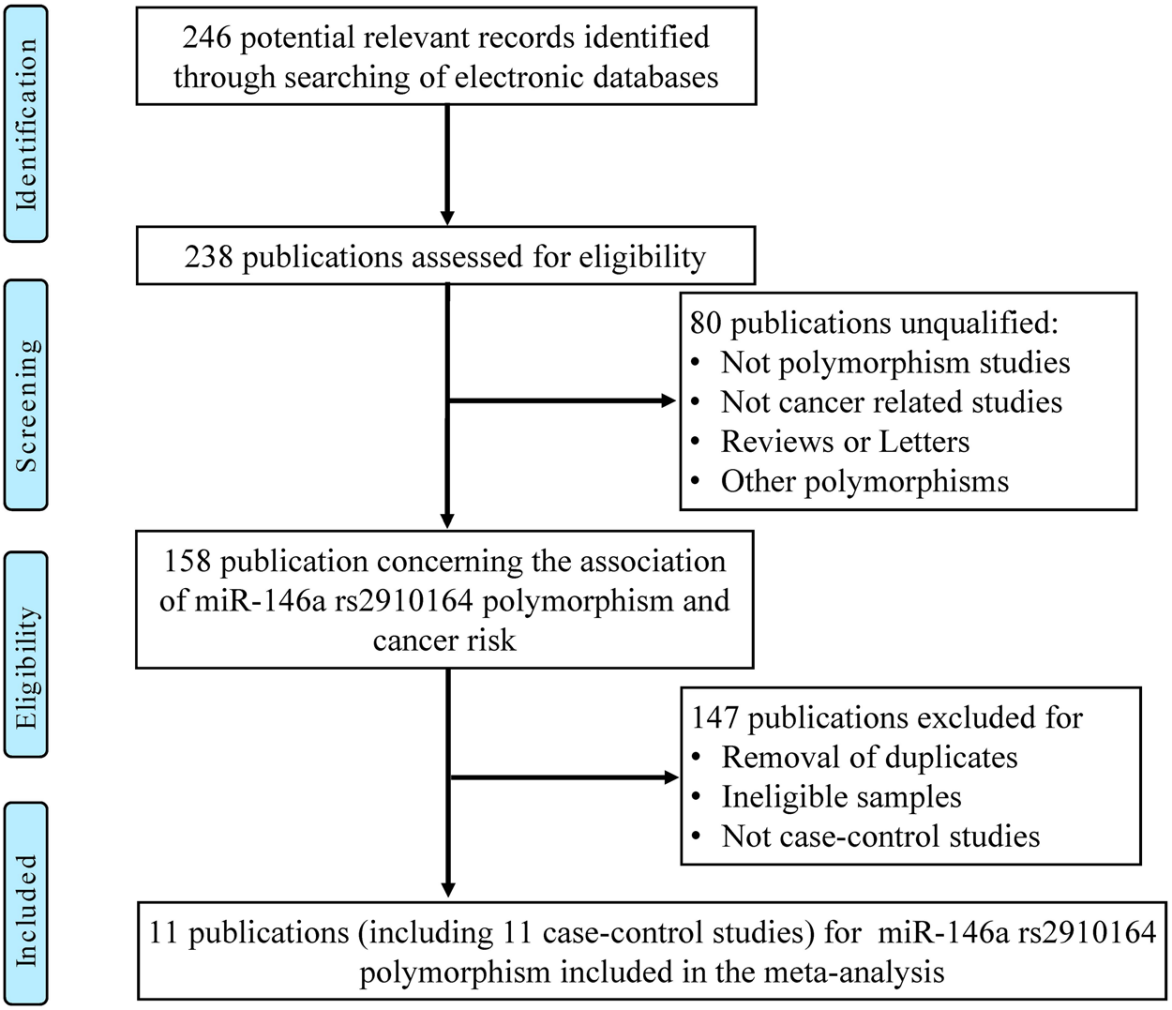
Flow chart of studies selection in this meta-analysis (miR-146a rs2910164).

**Figure 2:**
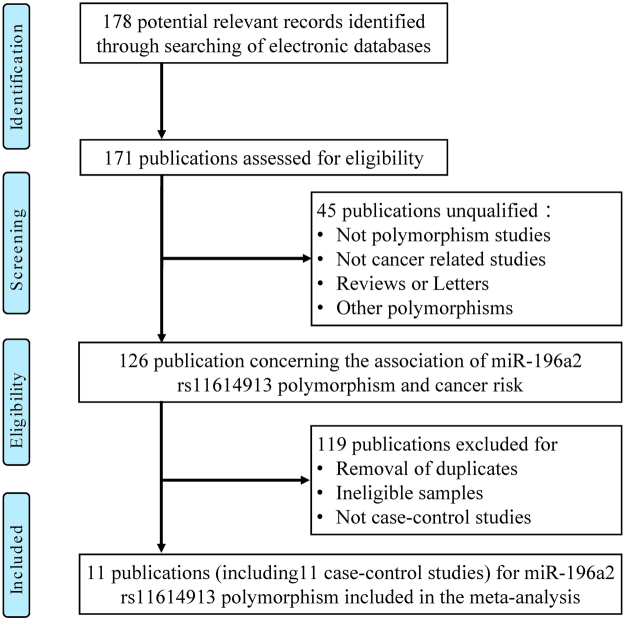
Flow chart of studies selection in this meta-analysis (miR-196a2 rs11614913).

**Figure 3:**
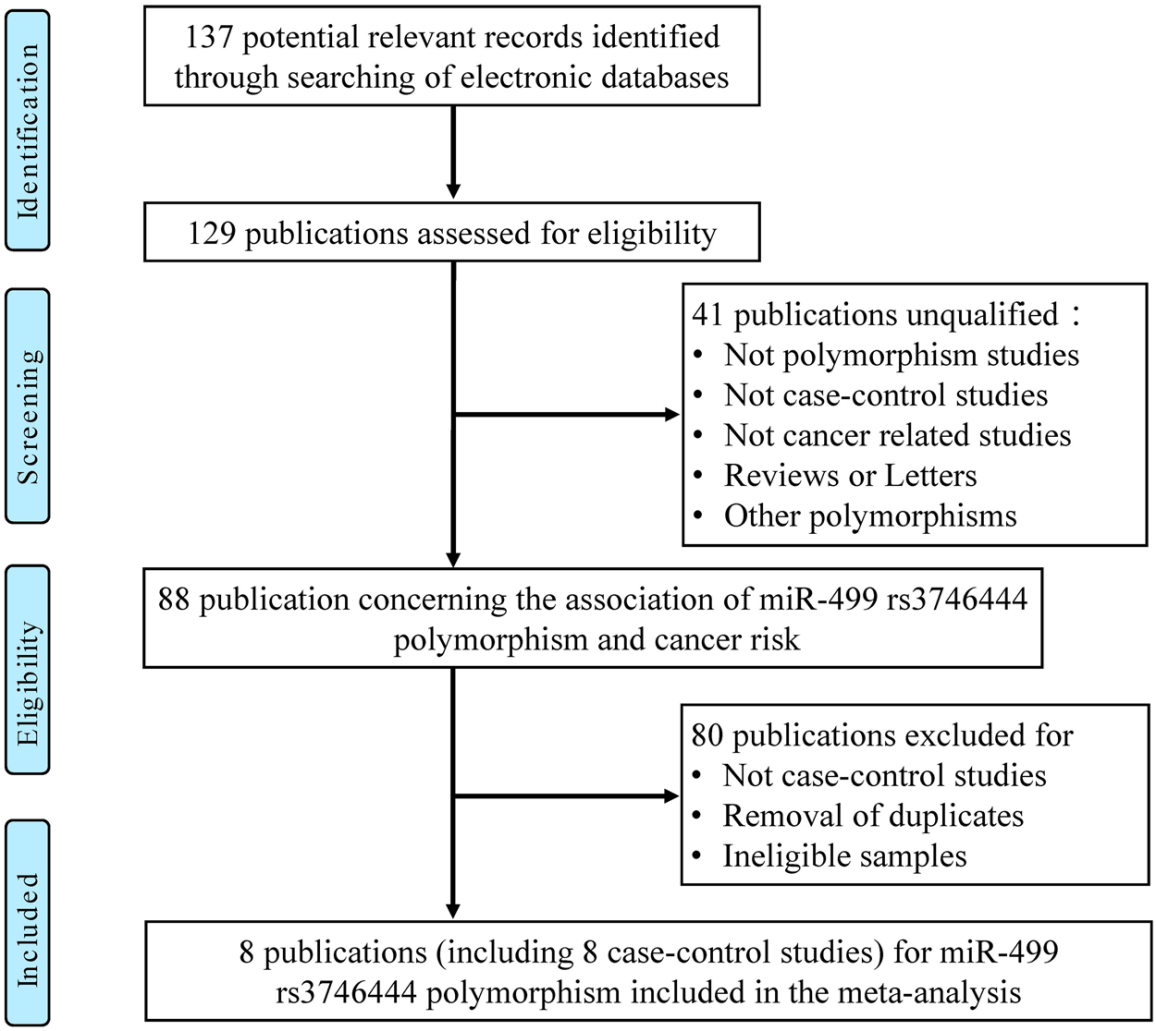
Flow chart of studies selection in this meta-analysis (miR-499 rs3746444).

Table 1 showed the characteristics of all the eligible studies and genotype frequency distributions of three miRNA polymorphisms (miRNA-146a rs2910164, miR-499 rs3746444 and miRNA-196a2 rs11614913) included in current meta-analysis^16,17,23–33^. The study selection processes were presented in Figs 1–3.

**Table 1:** Characteristics of the eligible studies included in the meta-analysis.

Gene (Polymorphism)	First Author	Year	Ethnicity	Source of Control	Cancer Type	Cases			Controls			
						AA	AB	BB	AA	AB	BB	HWE
miRNA-146a (rs2910164)	Mittal *et al*.	2011	Asian	H-B	BCa	6	79	127	7	108	135	N
	Yang *et al*.	2008	Caucasian	P-B	BCa	35	242	414	31	258	385	Y
	Wang *et al*.	2012	Asian	H-B	BCa	192	456	369	268	571	340	Y
	Deng *et al*.	2016	Asian	H-B	BCa	60	73	26	112	154	32	Y
	Horikawa *et al*.	2008	Caucasian	P-B	RCC	14	103	144	15	94	126	Y
	Du *et al*.	2014	Asian	P-B	RCC	118	167	68	115	190	57	Y
	Nikolic *et al*.	2014	Caucasian	P-B	PCa	12	90	184	7	63	129	Y
	Parlayan *et al*.	2014	Asian	H-B	PCa	37	41	11	216	237	71	Y
	Hashemi *et al*.	2016	Asian	H-B	PCa	13	131	25	11	147	24	Y
	George *et al*.	2011	Asian	P-B	PCa	76	79	4	116	107	7	N
	Xu *et al*.	2010	Asian	H-B	PCa	48	135	68	76	150	54	Y
miR-499 (rs3746444)	Mittal *et al*.	2011	Asian	H-B	BCa	95	92	25	121	94	35	Y
	Deng *et al*.	2015	Asian	P-B	BCa	107	45	7	216	68	14	Y
	Hu *et al*.	2009	Asian	P-B	BCa	707	258	44	816	248	29	N
	Du *et al*.	2014	Asian	P-B	RCC	251	94	9	255	96	11	Y
	Toraih *et al*.	2016	African	P-B	RCC	57	66	27	28	23	9	Y
	George *et al*.	2011	Asian	H-B	PCa	48	98	13	104	92	34	N
	Nikolic *et al*.	2015	Caucasian	H-B	PCa	190	147	18	180	110	17	Y
	Hashemi *et al*.	2016	Asian	H-B	PCa	62	82	25	85	64	33	Y
	Ginu *et al*.	2010	Asian	H-B	PCa	104	92	34	48	98	13	Y
miRNA-196a2 (rs11614913)	Mittal *et al*.	2012	Caucasian	P-B	BCa	76	131	5	109	127	14	N
	Deng *et al*.	2015	Asian	H-B	BCa	52	66	41	76	166	56	N
	Ma *et al*.	2013	Caucasian	P-B	BCa	255	348	133	257	342	132	Y
	George *et al*.	2011	Caucasian	H-B	PCa	55	101	3	106	114	10	N
	Nikoli *et al*.	2015	Caucasian	H-B	PCa	150	161	40	266	303	92	Y
	Hashemi *et al*.	2016	Asian	H-B	PCa	64	88	17	77	93	12	N
	Ma *et al*.	2013	Caucasian	P-B	RCC	105	126	45	101	117	59	N
	Toraih *et al*.	2016	Asian	H-B	RCC	23	31	11	80	53	17	Y
	Du *et al*.	2014	Asian	H-B	RCC	337	514	149	314	497	211	Y

A: wild allele; B: mutated allele; RCC: renal cell carcinoma; PCa: prostate cancer; BCa: bladder cancer; HWE: Hardy Weinberg Equilibrium; H-B: hospital-based; P-B: population-based; Y: study conformed to HWE; N: study did not conform to HWE.

For miRNA-146a rs2910164 polymorphism, a total of eleven studies with 3,647 cases and 4,413 controls met inclusion criteria. Eight of them were performed in Asian ethnicities and three in Caucasian ethnicities^16,17,19,23–26,28–30,32^. Controls of six studies were hospital-based (H-B), while five studies were population-based (P-B). Additionally, genotype frequencies of control groups were consistent with HWE, except for two studies^30,32^ (Table 1).

For miR-499 rs3746444 polymorphism, nine eligible studies with a total of 2,797 cases and 2,941 controls were enrolled^16,17,23,27,29,30,32,33^. Seven of them were performed on Asian ethnicities, one in Caucasian and African ethnicity, respectively. In addition, controls of five studies were H-B, and four were P-B. In addition, control groups of two studies were not consistent with HWE^30,32^.

For miRNA-196a2 rs11614913, we analyzed nine studies comprising 4,963 cases and 5,066 controls published between 2008 and July 2016^17,23,27,29,30,33–35^. Four studies were Asian ethnicities and rest were Caucasian ethnicities. In addition, three studies were conducted based on P-B, and the remaining six studies were H-B. As for HWE status, genotype distributions of control groups in five studies were not consistent with HWE^16,23,29,30,32^.

### Quantitative Data Synthesis

All the calculated results were summarized in Table 2.

**Table 2:** Results of the association between miRNA polymorphisms and urological neoplasms risk.

SNP	Comparison	Subgroup	N	*P*H	*P*Z	*P*A	Random	Fixed
rs11614913	B vs. A	Overall	9	0.019	0.850	1.000	1.011 (0.901–1.135)	0.961 (0.897–1.030)
	B vs. A	Asian	4	0.005	0.564	1.000	1.083 (0.826–1.421)	0.928 (0.837–1.030)
	B vs. A	Caucasian	5	0.310	0.812	1.000	0.991 (0.892–1.101)	0.989 (0.901–1.085)
	B vs. A	H-B	6	0.007	0.606	1.000	1.048 (0.876–1.254)	0.943 (0.864–1.029)
	B vs. A	P-B	3	0.411	0.907	1.000	0.993 (0.886–1.113)	0.993 (0.886–1.113)
	B vs. A	N	5	0.371	0.474	1.000	1.048 (0.922–1.192)	1.046 (0.924–1.185)
	B vs. A	Y	4	0.009	0.843	1.000	0.982 (0.818–1.179)	0.925 (0.851–1.006)
	B vs. A	BCa	3	0.824	0.681	1.000	1.025 (0.912–1.152)	1.025 (0.912–1.152)
	B vs. A	PCa	3	0.124	0.773	1.000	1.064 (0.853–1.329)	1.021 (0.884–1.180)
	B vs. A	RCC	3	0.009	0.992	1.000	1.002 (0.739–1.358)	0.881 (0.791–0.981)
	BA vs. AA	Overall	9	0.009	0.327	1.000	1.095 (0.913–1.313)	1.052 (0.947–1.169)
	BA vs. AA	Asian	4	0.015	0.935	1.000	1.016 (0.696–1.484)	0.971 (0.826–1.141)
	BA vs. AA	Caucasian	5	0.087	0.165	1.000	1.157 (0.941–1.423)	1.116 (0.972–1.282)
	BA vs. AA	H-B	6	0.005	0.573	1.000	1.082 (0.822–1.424)	1.020 (0.894–1.164)
	BA vs. AA	P-B	3	0.250	0.241	1.000	1.126 (0.909–1.394)	1.109 (0.933–1.319)
	BA vs. AA	N	5	0.007	0.505	1.000	1.125 (0.796–1.589)	1.145 (0.955–1.374)
	BA vs. AA	Y	4	0.168	0.898	1.000	1.030 (0.860–1.233)	1.008 (0.887–1.147)
	BA vs. AA	BCa	3	0.008	0.918	1.000	0.977 (0.629–1.517)	1.019 (0.852–1.219)
	BA vs. AA	PCa	3	0.069	0.328	1.000	1.193 (0.837–1.701)	1.131 (0.922–1.387)
	BA vs. AA	RCC	3	0.092	0.465	1.000	1.130 (0.814–1.569)	1.030 (0.871–1.218)
	BA + BB vs. AA	Overall	9	0.011	0.462	1.000	1.066 (0.899–1.265)	1.008 (0.913–1.114)
	BA + BB vs. AA	Asian	4	0.015	0.800	1.000	1.047 (0.733–1.496)	0.938 (0.805–1.093)
	BA + BB vs. AA	Caucasian	5	0.095	0.358	1.000	1.096 (0.902–1.331)	1.064 (0.932–1.213)
	BA + BB vs. AA	H-B	6	0.005	0.565	1.000	1.079 (0.832–1.399)	0.978 (0.863–1.109)
	BA + BB vs. AA	P-B	3	0.280	0.474	1.000	1.069 (0.881–1.297)	1.062 (0.901–1.251)
	BA + BB vs. AA	N	5	0.040	0.429	1.000	1.119 (0.846–1.481)	1.118 (0.938–1.332)
	BA + BB vs. AA	Y	4	0.047	0.937	1.000	1.009 (0.811–1.254)	0.960 (0.850–1.083)
	BA + BB vs. AA	BCa	3	0.064	0.940	1.000	1.012 (0.738–1.389)	1.025 (0.865–1.216)
	BA + BB vs. AA	PCa	3	0.060	0.381	1.000	1.171 (0.822–1.667)	1.094 (0.899–1.332)
	BA + BB vs. AA	RCC	3	0.025	0.681	1.000	1.084 (0.737–1.596)	0.943 (0.805–1.104)
	BB vs. AA	Overall	9	0.059	0.325	1.000	0.887 (0.699–1.126)	0.837 (0.721–0.973)
	BB vs. AA	Asian	4	0.008	0.620	1.000	1.153 (0.656–2.027)	0.821 (0.661–1.020)
	BB vs. AA	Caucasian	5	0.533	0.132	1.000	0.856 (0.694–1.055)	0.852 (0.692–1.049)
	BB vs. AA	H-B	6	0.033	0.829	1.000	0.961 (0.667–1.384)	0.804 (0.665–0.973)
	BB vs. AA	P-B	3	0.297	0.372	1.000	0.872 (0.648–1.173)	0.895 (0.701–1.142)
	BB vs. AA	N	5	0.287	0.438	1.000	0.897 (0.627–1.283)	0.888 (0.659–1.198)
	BB vs. AA	Y	4	0.020	0.557	1.000	0.900 (0.632–1.281)	0.821 (0.690–0.976)
	BB vs. AA	BCa	3	0.452	0.901	1.000	0.988 (0.768–1.272)	0.984 (0.766–1.265)
	BB vs. AA	PCa	3	0.190	0.483	1.000	0.939 (0.533–1.654)	0.881 (0.618–1.256)
	BB vs. AA	RCC	3	0.034	0.624	1.000	0.878 (0.522–1.478)	0.725 (0.582–0.904)
	BB vs. BA + AA	Overall	9	0.011	0.409	1.000	0.899 (0.699–1.157)	0.848 (0.741–0.971)
	BB vs. BA + AA	Asian	4	0.002	0.548	1.000	1.188 (0.677–2.083)	0.856 (0.706–1.038)
	BB vs. BA + AA	Caucasian	5	0.271	0.073	1.000	0.811 (0.637–1.032)	0.841 (0.696–1.017)
	BB vs. BA + AA	H-B	6	0.008	0.964	1.000	0.991 (0.677–1.452)	0.832 (0.701–0.988)
	BB vs. BA + AA	P-B	3	0.145	0.237	1.000	0.803 (0.551–1.169)	0.876 (0.703–1.091)
	BB vs. BA + AA	N	5	0.025	0.666	1.000	0.892 (0.531–1.498)	0.951 (0.727–1.245)
	BB vs. BA + AA	Y	4	0.057	0.298	1.000	0.862 (0.651–1.140)	0.816 (0.698–0.954)
	BB vs. BA + AA	BCa	3	0.059	0.969	1.000	1.010 (0.621–1.642)	1.048 (0.838–1.310)
	BB vs. BA + AA	PCa	3	0.158	0.395	1.000	0.896 (0.502–1.599)	0.865 (0.619–1.209)
	BB vs. BA + AA	RCC	3	0.141	0.001	**0.015**	0.778 (0.549–1.103)	0.716 (0.588–0.872)
rs2910164	B vs. A	Overall	11	0.497	0.000	**0.000**	1.140 (1.065–1.220)	1.140 (1.065–1.220)
	B vs. A	Asian	8	0.413	0.000	**0.000**	1.169 (1.079–1.266)	1.171 (1.083–1.266)
	B vs. A	Caucasian	3	0.852	0.497	1.000	1.049 (0.915–1.202)	1.049 (0.915–1.202)
	B vs. A	H-B	6	0.405	0.000	**0.000**	1.203 (1.100–1.316)	1.205 (1.104–1.315)
	B vs. A	P-B	5	0.988	0.407	1.000	1.047 (0.940–1.166)	1.047 (0.940–1.166)
	B vs. A	N	2	0.621	0.322	1.000	1.119 (0.895–1.399)	1.119 (0.896–1.398)
	B vs. A	Y	9	0.334	0.000	**0.000**	1.131 (1.045–1.224)	1.142 (1.063–1.226)
	B vs. A	BCa	4	0.490	0.000	**0.000**	1.186 (1.085–1.298)	1.186 (1.085–1.298)
	B vs. A	PCa	5	0.295	0.169	1.000	1.089 (0.939–1.263)	1.097 (0.961–1.253)
	B vs. A	RCC	2	0.871	0.575	1.000	1.050 (0.886–1.243)	1.050 (0.886–1.243)
	BA vs. AA	Overall	11	0.801	0.679	1.000	1.027 (0.902–1.170)	1.028 (0.903–1.170)
	BA vs. AA	Asian	8	0.638	0.544	1.000	1.043 (0.909–1.197)	1.043 (0.910–1.197)
	BA vs. AA	Caucasian	3	0.756	0.629	1.000	0.908 (0.612–1.346)	0.908 (0.613–1.344)
	BA vs. AA	H-B	6	0.624	0.346	1.000	1.081 (0.918–1.271)	1.081 (0.919–1.272)
	BA vs. AA	P-B	5	0.801	0.573	1.000	0.940 (0.758–1.166)	0.940 (0.758–1.166)
	BA vs. AA	N	2	0.650	0.658	1.000	1.091 (0.742–1.604)	1.091 (0.742–1.604)
	BA vs. AA	Y	9	0.664	0.779	1.000	1.019 (0.888–1.170)	1.020 (0.889–1.170)
	BA vs. AA	BCa	4	0.624	0.809	1.000	1.022 (0.853–1.225)	1.023 (0.853–1.225)
	BA vs. AA	PCa	5	0.629	0.335	1.000	1.122 (0.887–1.418)	1.122 (0.888–1.417)
	BA vs. AA	RCC	2	0.466	0.491	1.000	0.899 (0.663–1.218)	0.899 (0.663–1.218)
	BA + BB vs. AA	Overall	11	0.633	0.078	1.000	1.116 (0.986–1.262)	1.117 (0.988–1.263)
	BA + BB vs. AA	Asian	8	0.452	0.053	0.795	1.136 (0.997–1.294)	1.137 (0.998–1.295)
	BA + BB vs. AA	Caucasian	3	0.782	0.828	1.000	0.959 (0.657–1.402)	0.959 (0.657–1.400)
	BA + BB vs. AA	H-B	6	0.452	0.021	0.315	1.197 (1.026–1.395)	1.198 (1.027–1.396)
	BA + BB vs. AA	P-B	5	0.911	0.865	1.000	0.982 (0.798–1.209)	0.982 (0.799–1.208)
	BA + BB vs. AA	N	2	0.846	0.636	1.000	1.096 (0.750–1.603)	1.096 (0.750–1.603)
	BA + BB vs. AA	Y	9	0.442	0.090	1.000	1.118 (0.982–1.274)	1.119 (0.983–1.274)
	BA + BB vs. AA	BCa	4	0.514	0.096	1.000	1.155 (0.974–1.370)	1.156 (0.975–1.370)
	BA + BB vs. AA	PCa	5	0.398	0.224	1.000	1.148 (0.913–1.444)	1.150 (0.918–1.442)
	BA + BB vs. AA	RCC	2	0.530	0.802	1.000	0.964 (0.722–1.286)	0.964 (0.722–1.286)
	BB vs. AA	Overall	11	0.521	0.000	**0.000**	1.330 (1.137–1.555)	1.328 (1.137–1.552)
	BB vs. AA	Asian	8	0.537	0.000	**0.000**	1.409 (1.187–1.672)	1.407 (1.187–1.669)
	BB vs. AA	Caucasian	3	0.801	0.969	1.000	0.993 (0.676–1.459)	0.992 (0.676–1.457)
	BB vs. AA	H-B	6	0.481	0.000	**0.000**	1.476 (1.222–1.782)	1.474 (1.222–1.778)
	BB vs. AA	P-B	5	0.936	0.718	1.000	1.054 (0.795–1.396)	1.053 (0.795–1.394)
	BB vs. AA	N	2	0.789	0.984	1.000	0.992 (0.430–2.290)	0.991 (0.431–2.280)
	BB vs. AA	Y	9	0.381	0.000	**0.000**	1.326 (1.116–1.576)	1.343 (1.146–1.573)
	BB vs. AA	BCa	4	0.403	0.001	**0.015**	1.395 (1.144–1.702)	1.394 (1.144–1.700)
	BB vs. AA	PCa	5	0.240	0.161	1.000	1.177 (0.769–1.803)	1.270 (0.909–1.776)
	BB vs. AA	RCC	2	0.908	0.398	1.000	1.177 (0.806–1.720)	1.177 (0.806–1.720)
	BB vs. BA + AA	Overall	11	0.592	0.000	**0.000**	1.244 (1.123–1.377)	1.243 (1.123–1.376)
	BB vs. BA + AA	Asian	8	0.832	0.000	**0.000**	1.355 (1.191–1.542)	1.354 (1.190–1.540)
	BB vs. BA + AA	Caucasian	3	0.825	0.359	1.000	1.081 (0.916–1.275)	1.081 (0.916–1.275)
	BB vs. BA + AA	H-B	6	0.734	0.000	**0.000**	1.374 (1.197–1.577)	1.373 (1.196–1.575)
	BB vs. BA + AA	P-B	5	0.877	0.199	1.000	1.104 (0.949–1.284)	1.104 (0.949–1.284)
	BB vs. BA + AA	N	2	0.510	0.257	1.000	1.228 (0.861–1.752)	1.227 (0.861–1.749)
	BB vs. BA + AA	Y	9	0.439	0.000	**0.000**	1.245 (1.120–1.385)	1.244 (1.119–1.384)
	BB vs. BA + AA	BCa	4	0.370	0.000	**0.000**	1.295 (1.136–1.477)	1.295 (1.142–1.469)
	BB vs. BA + AA	PCa	5	0.464	0.249	1.000	1.146 (0.909–1.445)	1.145 (0.910–1.441)
	BB vs. BA + AA	RCC	2	0.498	0.275	1.000	1.156 (0.891–1.502)	1.157 (0.891–1.502)
rs3746444	B vs. A	Overall	9	0.422	0.010	0.150	1.125 (1.027–1.232)	1.126 (1.029–1.232)
	B vs. A	Asian	7	0.257	0.028	0.420	1.102 (0.982–1.238)	1.117 (1.012–1.233)
	B vs. A	Other ethnicities	2	0.630	0.161	1.000	1.169 (0.939–1.454)	1.169 (0.940–1.454)
	B vs. A	H-B	5	0.438	0.331	1.000	1.065 (0.938–1.208)	1.065 (0.938–1.208)
	B vs. A	P-B	4	0.418	0.007	0.105	1.192 (1.049–1.354)	1.192 (1.049–1.354)
	B vs. A	N	2	0.733	0.002	**0.030**	1.253 (1.083–1.450)	1.253 (1.084–1.450)
	B vs. A	Y	7	0.591	0.366	1.000	1.054 (0.940–1.181)	1.054 (0.940–1.181)
	B vs. A	BCa	3	0.444	0.007	0.105	1.199 (1.050–1.370)	1.199 (1.051–1.370)
	B vs. A	PCa	4	0.295	0.327	1.000	1.073 (0.915–1.257)	1.074 (0.931–1.238)
	B vs. A	RCC	2	0.285	0.691	1.000	1.056 (0.814–1.370)	1.049 (0.828–1.330)
	BA vs. AA	Overall	9	0.000	0.135	1.000	1.215 (0.941–1.569)	1.196 (1.064–1.344)
	BA vs. AA	Asian	7	0.000	0.287	1.000	1.191 (0.863–1.644)	1.178 (1.037–1.339)
	BA vs. AA	Other ethnicities	2	0.773	0.081	1.000	1.293 (0.969–1.724)	1.292 (0.969–1.724)
	BA vs. AA	H-B	5	0.000	0.432	1.000	1.226 (0.737–2.038)	1.220 (1.023–1.455)
	BA vs. AA	P-B	4	0.655	0.040	0.600	1.178 (1.008–1.377)	1.178 (1.008–1.377)
	BA vs. AA	N	2	0.009	0.141	1.000	1.613 (0.853–3.052)	1.345 (1.120–1.614)
	BA vs. AA	Y	7	0.001	0.500	1.000	1.114 (0.815–1.523)	1.103 (0.947–1.284)
	BA vs. AA	BCa	3	0.908	0.016	0.240	1.227 (1.039–1.449)	1.227 (1.039–1.449)
	BA vs. AA	PCa	4	0.000	0.556	1.000	1.221 (0.628–2.374)	1.213 (0.996–1.478)
	BA vs. AA	RCC	2	0.353	0.660	1.000	1.069 (0.794–1.439)	1.069 (0.794–1.438)
	BA + BB vs. AA	Overall	9	0.004	0.101	1.000	1.183 (0.968–1.447)	1.189 (1.064–1.329)
	BA + BB vs. AA	Asian	7	0.001	0.260	1.000	1.156 (0.898–1.489)	1.174 (1.039–1.326)
	BA + BB vs. AA	Other ethnicities	2	0.669	0.090	1.000	1.269 (0.964–1.670)	1.269 (0.964–1.670)
	BA + BB vs. AA	H-B	5	0.000	0.436	1.000	1.167 (0.791–1.720)	1.170 (0.989–1.385)
	BA + BB vs. AA	P-B	4	0.533	0.014	0.210	1.204 (1.038–1.397)	1.204 (1.038–1.397)
	BA + BB vs. AA	N	2	0.081	0.053	0.795	1.481 (0.994–2.206)	1.351 (1.135–1.609)
	BA + BB vs. AA	Y	7	0.014	0.468	1.000	1.095 (0.857–1.398)	1.088 (0.941–1.258)
	BA + BB vs. AA	BCa	3	0.910	0.007	0.105	1.242 (1.061–1.453)	1.242 (1.061–1.453)
	BA + BB vs. AA	PCa	4	0.000	0.547	1.000	1.169 (0.703–1.946)	1.174 (0.971–1.419)
	BA + BB vs. AA	RCC	2	0.279	0.676	1.000	1.080 (0.779–1.495)	1.063 (0.800–1.411)
	BB vs. AA	Overall	9	0.701	0.247	1.000	1.139 (0.908–1.428)	1.141 (0.912–1.428)
	BB vs. AA	Asian	7	0.536	0.309	1.000	1.134 (0.885–1.455)	1.136 (0.889–1.453)
	BB vs. AA	Other ethnicities	2	0.501	0.577	1.000	1.162 (0.674–2.004)	1.166 (0.679–2.004)
	BB vs. AA	H-B	5	0.960	0.919	1.000	0.985 (0.734–1.322)	0.985 (0.735–1.321)
	BB vs. AA	P-B	4	0.455	0.056	0.840	1.404 (0.986–1.998)	1.405 (0.991–1.992)
	BB vs. AA	N	2	0.091	0.522	1.000	1.268 (0.613–2.622)	1.392 (0.940–2.061)
	BB vs. AA	Y	7	0.976	0.792	1.000	1.036 (0.787–1.363)	1.037 (0.790–1.363)
	BB vs. AA	BCa	3	0.200	0.136	1.000	1.243 (0.785–1.967)	1.294 (0.922–1.816)
	BB vs. AA	PCa	4	0.913	0.943	1.000	1.013 (0.719–1.426)	1.012 (0.720–1.423)
	BB vs. AA	RCC	2	0.372	0.724	1.000	1.113 (0.594–2.087)	1.118 (0.602–2.079)
	BB vs. BA + AA	Overall	9	0.092	0.941	1.000	1.011 (0.756–1.352)	1.034 (0.836–1.277)
	BB vs. BA + AA	Asian	7	0.038	0.985	1.000	0.997 (0.691–1.438)	1.033 (0.819–1.302)
	BB vs. BA + AA	Other ethnicities	2	0.568	0.891	1.000	1.034 (0.612–1.748)	1.037 (0.616–1.747)
	BB vs. BA + AA	H-B	5	0.091	0.569	1.000	0.891 (0.600–1.324)	0.888 (0.677–1.164)
	BB vs. BA + AA	P-B	4	0.471	0.116	1.000	1.316 (0.930–1.863)	1.317 (0.934–1.857)
	BB vs. BA + AA	N	2	0.005	0.931	1.000	0.950 (0.299–3.023)	1.105 (0.760–1.607)
	BB vs. BA + AA	Y	7	0.483	0.992	1.000	0.991 (0.764–1.287)	1.001 (0.774–1.295)
	BB vs. BA + AA	BCa	3	0.137	0.301	1.000	1.138 (0.688–1.880)	1.190 (0.855–1.657)
	BB vs. BA + AA	PCa	4	0.047	0.740	1.000	0.915 (0.540–1.548)	0.911 (0.667–1.244)
	BB vs. BA + AA	RCC	2	0.517	0.904	1.000	1.035 (0.565–1.895)	1.038 (0.571–1.887)

*P*H: *P* value of heterogeneity; *P*Z: *P* value of Z test; *P*A: adjusted *P* value; A: wild allele; B: mutated allele; RCC: renal cell carcinoma; PCa: prostate cancer; BCa: bladder cancer; HWE: Hardy Weinberg Equilibrium; H-B: hospital-based; P-B: population-based; Y: study conformed to HWE; N: study did not conform to HWE.

#### miR-146a rs2910164

Overall analysis suggested significant associations between miR-146a rs2910164 polymorphism and urological neoplasms risk in allelic (B vs. A: OR = 1.140, 95% CI: 1.065–1.220, *P*_A_ < 0.001), homozygote (BB vs. AA: OR = 1.328, 95% CI: 1.137–1.552, *P*_A_ < 0.001) and recessive models (BB vs. BA + AA: OR = 1.243, 95% CI: 1.123–1.376, *P*_A_ < 0.001). Furthermore, in the stratified analysis by ethnicity, we uncovered significant associations between rs2910164 polymorphism and risk of urological neoplasms in Asian populations rather Caucasian in allelic (B vs. A: OR = 1.171, 95% CI: 1.083–1.226, *P*_A_ < 0.001), homozygote (BB vs. AA: OR = 1.407, 95% CI: 1.187–1.669, *P*_A_ < 0.001) and recessive models (BB vs. BA + AA: OR = 1.354, 95% CI: 1.190–1.540, *P*_A_ < 0.001). More importantly, when stratified analysis was conducted by cancer type, rs2910164 polymorphism was significantly associated with an increased risk of BCa in allelic (B vs. A: OR = 1.186, 95% CI: 1.085–1.298, *P*_A_ < 0.001), homozygote (BB vs. AA: OR = 1.394, 95% CI: 1.144–1.700, *P*_A_ < 0.001) and recessive models (BB vs. BA + AA: OR = 1.295, 95% CI: 1.142–1.469, *P*_A_ < 0.001). As for subgroup analyses by source of control and HWE status, we also found significant associations between H-B and HWE (Y) subgroups and risk of urological neoplasms in allelic, homozygote and recessive models, respectively.

#### miR-196a2 rs11614913

Overall, no significant association was uncovered for rs11614913 polymorphism and urological neoplasms risk. However, when subgroup analysis was conducted by cancer type, a significantly decreased risk of RCC was identified in recessive model (BB vs. BA + AA: OR = 0.716, 95% CI: 0.588–0.872, *P*_A_ = 0.015). While stratification analyses were conducted by source of control, ethnicity and HWE status, null result was uncovered (Table 2**)**.

#### miR-499 rs3746444

Overall, no significant association was uncovered for rs3746444 polymorphism and risk of urological neoplasms. Moreover, in the stratification analyses by cancer type, source of control and ethnicity, also null results were uncovered. Similarly, when subgroup analysis was conducted by HWE status, after excluding two studies that were not in accordance with HWE^30,32^, overall results were not changed (Table 2).

### Sensitivity Analysis

We repeat this meta-analysis and omit every study one by one, in order to examine effects of all the eligible studies involved in our study. The results showed that there was no material alteration in corresponding pooled ORs for miR-196a2 rs11614913 and miR-499 rs3746444 (Supplementary Table S2). For miR-146a rs2910164 polymorphism, the study conducted by Wang *et al*.^25^ was mainly considered as the reason of heterogeneity (Supplementary Table S2). After we excluded this study, there no longer existed heterogeneity, but still presented a negative association.

### Test of Heterogeneity

Both overall comparisons and subgroup analyses identified that heterogeneity was across the studies. Thus, we estimated source of heterogeneity within each model by performing further analyses stratified by source of control, ethnicity, HWE status and cancer type. For rs11614913 polymorphism, ethnicity (*P*_heterogeneity_ = 0.005), source of controls (*P*_heterogeneity_ = 0.007), HWE status (*P*_heterogeneity_ = 0.009) and cancer type (*P*_heterogeneity_ = 0.009) showed potential sources of between-study heterogeneity. For rs2910164 and rs3746444, no source was observed contributed to the substantial heterogeneity.

### Publication Bias

**Figure 4:**
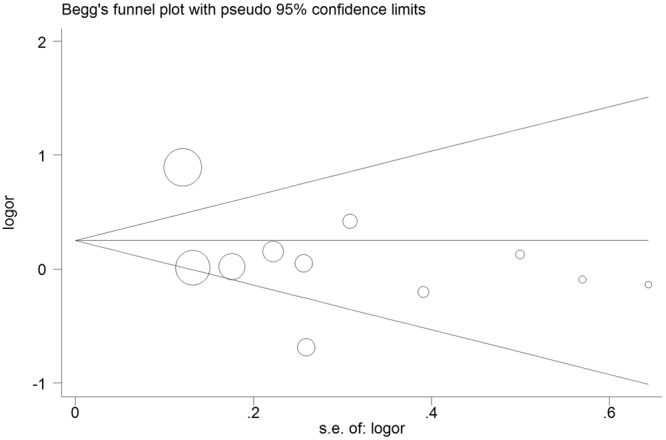
Begg’s funnel plot for publication bias test for miR-146a rs2910164 polymorphism. The x-axis is log (OR), and the y-axis is natural logarithm of OR. The horizontal line in the figure represents the overall estimated log (OR). The two diagonal lines indicate the pseudo 95% confidence limits of the effect estimate. Log (OR) = log-transformed OR, OR = odds ratio.

**Figure 5:**
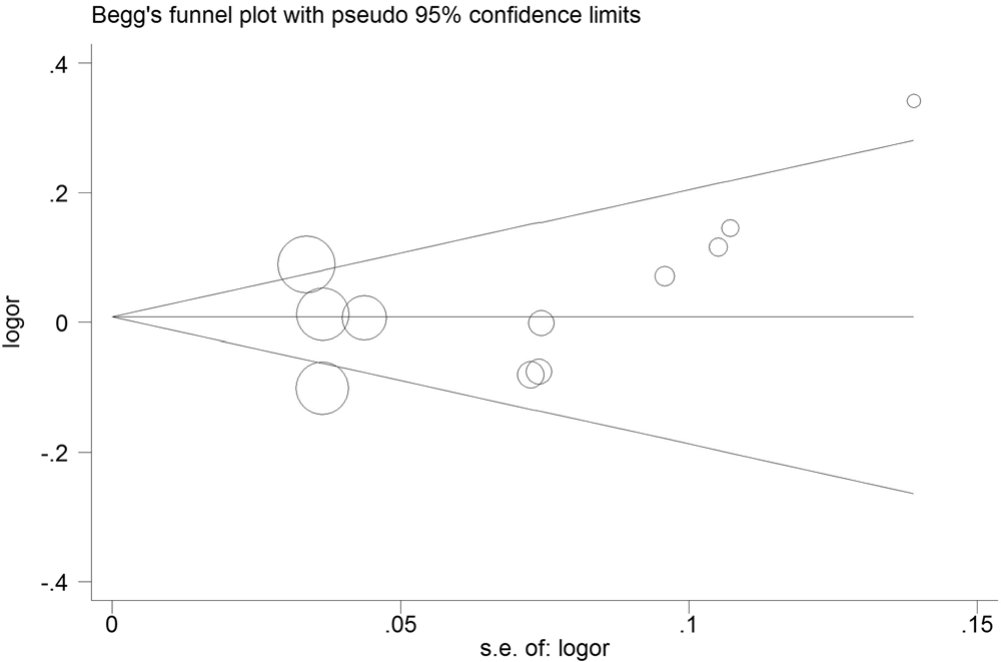
Begg’s funnel plot for publication bias test for miR-196a2 rs11614913 polymorphism. The x-axis is log (OR), and the y-axis is natural logarithm of OR. The horizontal line confidence limits of the effect estimate. Log (OR) = log-transformed OR, OR = odds ratio.

**Figure 6:**
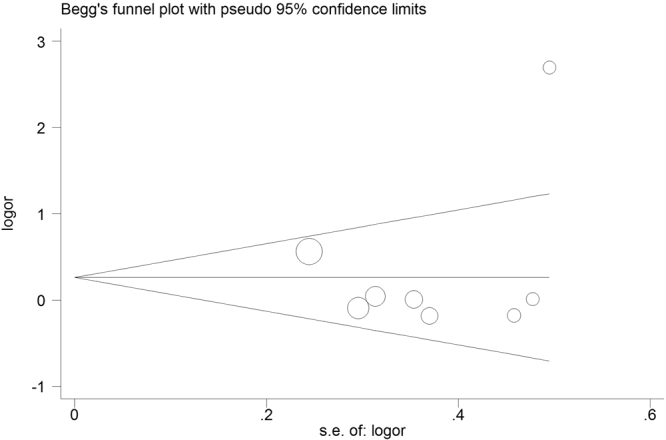
Begg’s funnel plot for publication bias test for miR-499 rs3746444 polymorphism. The x-axis is log (OR), and the y-axis is natural logarithm of OR. The horizontal line in the figure represents the overall estimated log (OR). The two diagonal lines indicate the pseudo 95% confidence limits of the effect estimate. Log (OR) = log-transformed OR, OR = odds ratio.

Both Begg’s funnel plot and Egger’s regression tests were conducted to evaluate potential publication bias. In the funnel diagram, if publication bias was not existed, data obtained from each study will be presented with an inverted funnel-like symmetric distribution on the graph, whereas asymmetric inverted funnel graph suggested publication bias. As for miR-146a rs2910164 and miR-499 rs3746444 polymorphisms, neither Begg’s funnel plot nor Egger’s regression showed evidence of publication bias (Shape of funnel plot was symmetrical, which was further confirmed by Egger’s regression test; Supplementary Table S3 and Figs 4–6**)**. For miR-196a2 rs11614913 polymorphism, publication bias was existed both in H-B and HWE (N) subgroups. Therefore, we further conducted sensitivity analyses using the trim and fill method^36^, and imputed studies provide a symmetrical funnel plot (data not shown), indicating that no publication bias was existed in both H-B and HWE (N) groups.

## Discussion

In human, miRNA genes are approximately 50% situated in genomic areas that are frequently related to tumor. Polymorphisms are the most common type of genetic factors within the human genome, which may cause differences of phenotype^37^; and such polymorphisms in miRNA may influence the formation of miRNAs, pri-miRNAs, pre-miRNAs and/or mature miRNAs, and/or the selection of targets and therefore obviously influence an individual’s cancer susceptibility^38^. Here, we assessed the relationship between three common miRNA variations (miR-146a rs2910164, miR-196a2 rs11614913 and miR-499 rs3746444) and their risk of urological neoplasms. Current study was a kind of stratified investigations based on large populations and various cancer types. Significant relationship between miR-146a rs2910164 polymorphism and urological neoplasms risk was found, particularly in Asians instead of Caucasians. Especially, in the stratification analysis by cancer type, rs2910164 polymorphism was significantly associated with an increased risk of BCa. Although for rs11614913 and rs3746444 polymorphisms, overall analyses uncovered negative results, however, for rs11614913 polymorphism, when subgroup analysis was conducted by cancer type, a significantly decreased risk of RCC was identified in a recessive model. In addition, as for Begg’s funnel plot and Egger’s test, the adjusted results indicated that publication bias was not existed.

Several investigators have paid attention to a single polymorphism or several polymorphisms in miRNA and cancer risk. Anyway, none of these meta-analyses have exhaustively enrolled all the available miRNA polymorphisms or commonly studied miRNA polymorphisms. In the current analysis, we covered the previously published eligible publications, and added some updated works. By comparing with previous meta-analysis works, several advantages in current study should be pointed. First of all, a more comprehensive analysis was conducted with large sample sizes, for the purpose of strengthening statistical power and reliability of conclusions. Secondly, we performed various subgroup analysis by ethnicity, source of controls and so on, in order to provide the sources of heterogeneity and the tumor and/or race markers. Thirdly, data in our work was adjusted referring to a commonly used formula in genome-wide association study (GWAS); our conclusions suggested that miR-146a rs2910164 polymorphism was associated with an increased risk of urological neoplasms, particularly for BCa in Asians, whereas in the study conducted by Xiao *et al*.^35^, they indicated that miR-146a rs2910164 B allele is associated with a decreased risk of BCa and PCa in Asians.

Nevertheless, there were still several limitations that should be noted in present work. Firstly, for miR-196a2 rs11614913 polymorphism, relatively heterogeneity existed between some studies, although we conducted this analysis with severe inclusion criteria and explicit extraction data. Therefore, after stratified analysis by ethnicity, source of control, HWE status and cancer type, we observed that the subgroup heterogeneity reduced significantly. It can be assumed that the heterogeneity possibly derived from difference of ethnicity, source of control, HWE status and cancer type. Secondly, the small sample sizes of studies may cause low power for data, particularly in subgroup analyses. Thirdly, we did not assess potential interactions due to lack of relevant data across the included studies; this is of extreme significance because interactions between gene-gene and gene-environment may modify kinds of disease risk. Last, our study was in lack of African ethnicities; therefore, more studies based on African populations were needed to be included in our analysis for drawing a more comprehensive conclusion.

In summary, our conclusions demonstrate that rs2910164 polymorphism is a risk factor for urological neoplasms in Asians, particularly for BCa. Further studies with larger sample sizes are warranted to clarify the possible roles of these polymorphisms in urological neoplasms.

## Electronic supplementary material

Supplementary Information

## References

[1] Ambros V (2004). The functions of animal microRNAs. Nature.

[2] Tahi F, Du TTV, Boucheham A (2017). In Silico Prediction of RNA Secondary Structure. Methods Mol Biol1.

[3] Ruan K, Fang X, Ouyang G (2009). MicroRNAs: novel regulators in the hallmarks of human cancer. Cancer letters.

[4] Kwak PB, Iwasaki S, Tomari Y (2010). The microRNA pathway and cancer. Cancer science.

[5] Qu KZ, Zhang K, Li H, Afdhal NH, Albitar M (2011). Circulating microRNAs as biomarkers for hepatocellular carcinoma. Journal of clinical gastroenterology.

[6] Komar AA (2007). Silent SNPs: impact on gene function and phenotype. Pharmacogenomics.

[7] Saunders MA, Liang H, Li W-H (2007). Human polymorphism at microRNAs and microRNA target sites. Proceedings of the National Academy of Sciences.

[8] Landi D, Gemignani F, Landi S (2012). Role of variations within microRNA-binding sites in cancer. Mutagenesis.

[9] Zhou F (2012). A functional polymorphism in Pre-miR-146a is associated with susceptibility to gastric cancer in a Chinese population. DNA and cell biology.

[10] Ryan BM, Robles AI, Harris CC (2010). Genetic variation in microRNA networks: the implications for cancer research. Nat Rev Cancer.

[11] Hoffman AE (2009). microRNA miR-196a-2 and breast cancer: a genetic and epigenetic association study and functional analysis. Cancer research.

[12] Hezova R (2012). Evaluation of SNPs in miR-196-a2, miR-27a and miR-146a as risk factors of colorectal cancer. World J Gastroenterol.

[13] Kim WH (2012). Association study of microRNA polymorphisms with hepatocellular carcinoma in Korean population. Gene.

[14] Srivastava K, Srivastava A, Mittal B (2010). Common genetic variants in pre-microRNAs and risk of gallbladder cancer in North Indian population. Journal of human genetics.

[15] Guo H (2010). A functional varient in microRNA-146a is associated with risk of esophageal squamous cell carcinoma in Chinese Han. Familial cancer.

[16] Deng S, Wang W, Li X, Zhang P (2015). Common genetic polymorphisms in pre-microRNAs and risk of bladder cancer. World J Surg Oncol.

[17] Du M (2014). Genetic variations in microRNAs and the risk and survival of renal cell cancer. Carcinogenesis.

[18] Ma XP, Zhang T, Peng B, Yu L (2013). & Jiang de, K. Association between microRNA polymorphisms and cancer risk based on the findings of 66 case-control studies. PLoS One.

[19] Xu B (2010). A functional polymorphism in Pre-miR-146a gene is associated with prostate cancer risk and mature miR-146a expression *in vivo* †. Prostate.

[20] Ammarin T, Patrick M, Catherine DE, David D, John A (2005). A method for meta-analysis of molecular association studies. Statistics in Medicine.

[21] Cochran WG (1968). The effectiveness of adjustment by subclassification in removing bias in observational studies. Biometrics.

[22] Mantel N, Haenszel W (1959). Statistical aspects of the analysis of data from retrospective studies of disease. J Natl Cancer Inst.

[23] Mittal RD, Gangwar R, George GP, Mittal T, Kapoor R (2011). Investigative role of pre-microRNAs in bladder cancer patients: a case-control study in North India. DNA Cell Biol.

[24] Yang H (2008). Evaluation of genetic variants in microRNA-related genes and risk of bladder cancer. Cancer Res.

[25] Wang M (2012). Genetic variants in miRNAs predict bladder cancer risk and recurrence. Cancer Res.

[26] Horikawa Y (2008). Single nucleotide polymorphisms of microRNA machinery genes modify the risk of renal cell carcinoma. Clin Cancer Res.

[27] Nikolic Z (2015). Assessment of association between genetic variants in microRNA genes hsa-miR-499, hsa-miR-196a2 and hsa-miR-27a and prostate cancer risk in Serbian population. Exp Mol Pathol.

[28] Parlayan C (2014). Association Analysis of Single Nucleotide Polymorphisms in miR-146a and miR-196a2 on the Prevalence of Cancer in Elderly Japanese: A Case-Control Study. Asian Pacific Journal of Cancer Prevention.

[29] Hashemi M (2016). Association between single nucleotide polymorphism in miR-499, miR-196a2, miR-146a and miR-149 and prostate cancer risk in a sample of Iranian population. Journal of Advanced Research.

[30] George GP, Gangwar R, Mandal RK, Sankhwar SN, Mittal RD (2011). Genetic variation in microRNA genes and prostate cancer risk in North Indian population. Mol Biol Rep.

[31] Xu B (2010). A functional polymorphism in Pre-miR-146a gene is associated with prostate cancer risk and mature miR-146a expression *in vivo*. Prostate.

[32] Hu M, Zhao L, Hu S, Yang J (2013). The Association between Two Common Polymorphisms in MicroRNAs and Hepatocellular Carcinoma Risk in Asian Population. Plos One.

[33] Toraih, E. A. *et al*. Combined Genotype Analyses of Precursor miRNA196a2 and 499a Variants with Hepatic and Renal Cancer Susceptibility a Preliminary Study. *Asian Pacific Journal of Cancer Prevention Apjcp* **17** (2016). 27509977

[34] Deng S, Wang W, Li X, Zhang P (2014). Common genetic polymorphisms in pre-microRNAs and risk of bladder cancer. World Journal of Surgical Oncology.

[35] Xiao PM, Zhang T, Bo P, Long Y, Jiang DK (2013). Association between microRNA Polymorphisms and Cancer Risk Based on the Findings of 66 Case-ControlStudies. Plos One.

[36] Sue D, Richard T (2000). Trim and Fill: A Simple Funnel-Plot–Based Method of Testing and Adjusting for Publication Bias in Meta-Analysis. Biometrics.

[37] Hinds DA (2005). Whole-Genome Patterns of Common DNA Variation in Three Human Populations. Science.

[38] Duan R, Pak CH, Jin P (2007). Single nucleotide polymorphism associated with mature miR-125a alters the processing of pri-miRNA. Human Molecular Genetics.

