# Supplementary material for: Comprehensive Review of Genetic Association Studies and Meta-Analysis on polymorphisms in microRNAs and Urological Neoplasms Risk
Source: Sci Rep. 2018 Feb 28;8:3776. doi: 10.1038/s41598-018-21749-4 (PMC5830532; doi:10.1038/s41598-018-21749-4)
Supplement: Supplementary file 1 — Supplementary Information [file 41598_2018_21749_MOESM1_ESM.docx]

**Comprehensive Review of Genetic Association Studies and Meta-Analysis on polymorphisms in microRNAs and Urological Neoplasms Risk**

Ligang Zhang^1,2#^, Meng Zhang^1,2#^, Hao Wang^1,2#^, Yangyang Wang^1,2^, Jun Zhou^1,2^, Zongyao Hao^1,2^, Li Zhang^1,2*^, Chaozhao Liang^1,2*^

^1^ Department of Urology, the First Affiliated Hospital of Anhui Medical University, Hefei 230022, China;

^2^ Institute of Urology, Anhui Medical University, Hefei 230022, China.

^#^ These authors contributed equally to the work.

*** Correspondence:**

Name: Chaozhao Liang;

^1^ Department of Urology, the First Affiliated Hospital of Anhui Medical University, Hefei 230022, China;

^2^ Institute of Urology, Anhui Medical University, Hefei 230022, China.

Tel.: +86 55162923440

Email adress: [liang_chaozhao@ahmu.edu.cn](mailto:liang_chaozhao@ahmu.edu.cn)

Name: Li Zhang

^1^ Department of Urology, the First Affiliated Hospital of Anhui Medical University, Hefei 230022, China;

^2^ Institute of Urology, Anhui Medical University, Hefei 230022, China.

Tel.: +86 55162923932

Email address: [lzhang@ahmu.edu.cn](mailto:lzhang@ahmu.edu.cn)

**Table S1.** Methodological quality of the included studies according to the Newcastle-Ottawa Scale.

| **Author** | **Ethnicity** | **Adequacy of Case Definition** | **Representativeness of the Cases** | **Selection of Controls** | **Definition of Controls** | **Comparability Cases/Controls** | **Ascertainment of Exposure** | **Same Method of Ascertainment** | **Non-response rate** |
| --- | --- | --- | --- | --- | --- | --- | --- | --- | --- |
| **Rs2910164** | | | | | | | | | |
| Mittal et al | Asian | * | * | NA | * | ** | * | * | * |
| Yang et al | Caucasian | * | * | NA | * | ** | * | * | * |
| Wang et al | Asian | * | * | NA | * | ** | * | * | * |
| Deng et al | Asian | * | * | * | NA | ** | * | * | * |
| Horikawa et al | Caucasian | * | * | NA | * | ** | * | * | * |
| Du et al | Asian | * | * | NA | NA | ** | * | * | * |
| Nikolic et al | Caucasian | * | * | NA | * | ** | * | * | * |
| Parlayan et al | Asian | * | * | NA | * | ** | * | * | * |
| Hashemi et al | Asian | * | * | NA | * | ** | * | * | * |
| George et al | Asian | * | * | NA | * | ** | * | * | * |
| **Rs3746444** | | | | | | | | | |
| Xu et al | Asian | * | * | * | * | ** | * | * | * |
| Mittal et al | Asian | * | * | NA | * | ** | * | * | * |
| Deng et al | Asian | * | * | * | NA | ** | * | * | * |
| Hu et al | Asian | * | * | NA | * | ** | * | * | * |
| Du et al | Asian | * | * | NA | NA | ** | * | * | * |
| Toraih et al | African | * | * | NA | * | ** | * | * | * |
| George et al | Asian | * | * | NA | * | ** | * | * | * |
| Nikolic et al | Caucasian | * | * | NA | * | ** | * | * | * |
| Hashemi et al | Asian | * | * | NA | * | ** | * | * | * |
| Ginu et al | Asian | * | * | * | * | ** | * | * | * |
| **Rs11614913** | | | | | | | | | |
| Mittal et al | Caucasian | * | * | NA | * | ** | * | * | * |
| Deng et al | Asian | * | * | * | NA | ** | * | * | * |
| Ma et al | Caucasian | * | * | NA | * | ** | * | * | * |
| George et al | Caucasian | * | * | NA | * | ** | * | * | * |
| Nikolic et al | Caucasian | * | * | NA | * | ** | * | * | * |
| Hashemi et al | Asian | * | * | NA | * | ** | * | * | * |
| Ma et al | Caucasian | * | * | NA | * | ** | * | * | * |
| Toraih et al | African | * | * | NA | * | ** | * | * | * |
| Du et al | Asian | * | * | NA | NA | ** | * | * | * |

This table identifies ‘high’ quality choices with a ‘star’. A study can be awarded a maximum of one star for each numbered item within the Selection and Exposure categories. A maximum of two stars can be given for Comparability.

**Table S2.** Details of the sensitivity analyses for the polymorphisms in miRNAs and urological neoplasm risk.

| **Polymorphism** | **Comparison** | **Study Omitted** | **Estimate** | **95%CI** | **Effect Model** |
| --- | --- | --- | --- | --- | --- |
| rs2910164 | B vs. A | Mittal *et al.* (2011) | 1.137 | 1.061-1.219 | Fixed |
|  |  | Yang *et al.* (2008) | 1.150 | 1.071-1.240 |  |
|  |  | Wang *et al.* (2012) | 1.087 | 1.001-1.181 |  |
|  |  | Deng *et al.* (2016) | 1.141 | 1.063-1.223 |  |
|  |  | Horikawa *et al.* (2008) | 1.144 | 1.067-1.223 |  |
|  |  | Du *et al.* (2014) | 1.152 | 1.072-1.238 |  |
|  |  | Nikolic *et al.* (2014) | 1.148 | 1.071-1.231 |  |
|  |  | Parlayan *et al.* (2014) | 1.148 | 1.071-1.230 |  |
|  |  | Hashemi *et al.* (2016) | 1.148 | 1.071-1.231 |  |
|  |  | George *et al.* (2011) | 1.144 | 1.067-1.226 |  |
|  |  | Xu *et al.* (2010) | 1.121 | 1.045-1.204 |  |
|  | BA vs. AA | Mittal *et al.* (2011) | 1.030 | 0.904-1.174 | Fixed |
|  |  | Yang *et al.* (2008) | 1.043 | 0.912-1.192 |  |
|  |  | Wang *et al.* (2012) | 0.986 | 0.841-1.156 |  |
|  |  | Deng *et al.* (2016) | 1.044 | 0.911-1.196 |  |
|  |  | Horikawa *et al.* (2008) | 1.024 | 0.898-1.167 |  |
|  |  | Du *et al.* (2014) | 1.062 | 0.923-1.223 |  |
|  |  | Nikolic *et al.* (2014) | 1.032 | 0.905-1.175 |  |
|  |  | Parlayan *et al.* (2014) | 1.029 | 0.900-1.178 |  |
|  |  | Hashemi *et al.* (2016) | 1.036 | 0.908-1.181 |  |
|  |  | George *et al.* (2011) | 1.017 | 0.888-1.166 |  |
|  |  | Xu *et al.* (2010) | 0.994 | 0.868-1.139 |  |
|  | BA+BB vs. AA | Mittal *et al.* (2011) | 1.118 | 0.988-1.266 | Fixed |
|  |  | Yang *et al.* (2008) | 1.132 | 0.997-1.286 |  |
|  |  | Wang *et al.* (2012) | 1.044 | 0.896-1.216 |  |
|  |  | Deng *et al.* (2016) | 1.131 | 0.993-1.287 |  |
|  |  | Horikawa *et al.* (2008) | 1.114 | 0.984-1.262 |  |
|  |  | Du *et al.* (2014) | 1.155 | 1.011-1.321 |  |
|  |  | Nikolic *et al.* (2014) | 1.122 | 0.991-1.271 |  |
|  |  | Parlayan *et al.* (2014) | 1.128 | 0.993-1.281 |  |
|  |  | Hashemi *et al.* (2016) | 1.126 | 0.994-1.275 |  |
|  |  | George *et al.* (2011) | 1.117 | 0.982-1.271 |  |
|  |  | Xu *et al.* (2010) | 1.079 | 0.948-1.227 |  |
|  | BB vs. AA | Mittal *et al.* (2011) | 1.333 | 1.140-1.560 | Fixed |
|  |  | Yang *et al.* (2008) | 1.376 | 1.169-1.621 |  |
|  |  | Wang *et al.* (2012) | 1.200 | 0.976-1.476 |  |
|  |  | Deng *et al.* (2016) | 1.316 | 1.121-1.546 |  |
|  |  | Horikawa *et al.* (2008) | 1.333 | 1.137-1.562 |  |
|  |  | Du *et al.* (2014) | 1.355 | 1.147-1.600 |  |
|  |  | Nikolic *et al.* (2014) | 1.346 | 1.149-1.576 |  |
|  |  | Parlayan *et al.* (2014) | 1.354 | 1.154-1.588 |  |
|  |  | Hashemi *et al.* (2016) | 1.343 | 1.147-1.572 |  |
|  |  | George *et al.* (2011) | 1.337 | 1.143-1.564 |  |
|  |  | Xu *et al.* (2010) | 1.273 | 1.081-1.499 |  |
|  | BB vs. AA+AB | Mittal *et al.* (2011) | 1.240 | 1.116-1.379 | Random |
|  |  | Yang *et al.* (2008) | 1.280 | 1.141-1.436 |  |
|  |  | Wang *et al.* (2012) | 1.173 | 1.037-1.327 |  |
|  |  | Deng *et al.* (2016) | 1.232 | 1.111-1.366 |  |
|  |  | Horikawa *et al.* (2008) | 1.260 | 1.133-1.401 |  |
|  |  | Du *et al.* (2014) | 1.240 | 1.116-1.378 |  |
|  |  | Nikolic *et al.* (2014) | 1.266 | 1.139-1.407 |  |
|  |  | Parlayan *et al.* (2014) | 1.252 | 1.130-1.388 |  |
|  |  | Hashemi *et al.* (2016) | 1.246 | 1.124-1.381 |  |
|  |  | George *et al.* (2011) | 1.246 | 1.126-1.380 |  |
|  |  | Xu *et al.* (2010) | 1.224 | 1.102-1.360 |  |
| rs11614913 | B vs. A | Mittal *et al.* (2011) | 1.003 | 0.886-1.136 | Random |
|  |  | Deng *et al.* (2015) | 1.018 | 0.895-1.156 |  |
|  |  | Ma *et al.* (2013) | 1.020 | 0.889-1.172 |  |
|  |  | George *et al.* (2011) | 0.990 | 0.881-1.113 |  |
|  |  | Nikoli *et al.* (2015) | 1.036 | 0.908-1.183 |  |
|  |  | Hashemi *et al.* (2016) | 0.996 | 0.883-1.122 |  |
|  |  | Ma *et al.* (2013) | 1.034 | 0.910-1.175 |  |
|  |  | Toraih *et al.* (2016) | 0.971 | 0.881-1.071 |  |
|  |  | Du *et al.* (2014) | 1.044 | 0.933-1.170 |  |
|  | BA vs. AA | Mittal *et al.* (2011) | 1.055 | 0.886-1.136 | Random |
|  |  | Deng *et al.* (2015) | 1.145 | 0.895-1.156 |  |
|  |  | Ma *et al.* (2013) | 1.115 | 0.889-1.172 |  |
|  |  | George *et al.* (2011) | 1.040 | 0.881-1.113 |  |
|  |  | Nikoli *et al.* (2015) | 1.126 | 0.908-1.388 |  |
|  |  | Hashemi *et al.* (2016) | 1.094 | 0.896-1.336 |  |
|  |  | Ma *et al.* (2013) | 1.107 | 0.902-1.358 |  |
|  |  | Toraih *et al.* (2016) | 0.971 | 0.886-1.252 |  |
|  |  | Du *et al.* (2014) | 1.104 | 0.906-1.405 |  |
|  | BA+BB vs. AA | Mittal *et al.* (2011) | 1.033 | 0.866-1.232 | Random |
|  |  | Deng *et al.* (2015) | 1.108 | 0.931-1.319 |  |
|  |  | Ma *et al.* (2013) | 1.085 | 0.882-1.335 |  |
|  |  | George *et al.* (2011) | 1.015 | 0.863-1.193 |  |
|  |  | Nikoli *et al.* (2015) | 1.101 | 0.905-1.341 |  |
|  |  | Hashemi *et al.* (2016) | 1.057 | 0.878-1.271 |  |
|  |  | Ma *et al.* (2013) | 1.090 | 0.900-1.321 |  |
|  |  | Toraih *et al.* (2016) | 1.016 | 0.871-1.186 |  |
|  |  | Du *et al.* (2014) | 1.112 | 0.915-1.352 |  |
|  | BB vs. AA+BA | Mittal *et al.* (2011) | 0.934 | 0.725-1.202 | Random |
|  |  | Deng *et al.* (2015) | 0.830 | 0.656-1.050 |  |
|  |  | Ma *et al.* (2013) | 0.882 | 0.701-1.244 |  |
|  |  | George *et al.* (2011) | 0.922 | 0.714-1.192 |  |
|  |  | Nikoli *et al.* (2015) | 0.920 | 0.685-1.236 |  |
|  |  | Hashemi *et al.* (2016) | 0.861 | 0.668-1.109 |  |
|  |  | Ma *et al.* (2013) | 0.933 | 0.701-1.243 |  |
|  |  | Toraih *et al.* (2016) | 0.864 | 0.670-1.114 |  |
|  |  | Du *et al.* (2014) | 0.964 | 0.736-1.262 |  |
|  | BB vs. AA | Mittal *et al.* (2011) | 0.911 | 0.711-1.167 | Random |
|  |  | Deng *et al.* (2015) | 0.869 | 0.667-1.132 |  |
|  |  | Ma *et al.* (2013) | 0.865 | 0.654-1.144 |  |
|  |  | George *et al.* (2011) | 0.902 | 0.702-1.158 |  |
|  |  | Nikoli *et al.* (2015) | 0.918 | 0.693-1.216 |  |
|  |  | Hashemi *et al.* (2016) | 0.843 | 0.671-1.061 |  |
|  |  | Ma *et al.* (2013) | 0.921 | 0.700-1.211 |  |
|  |  | Toraih *et al.* (2016) | 0.832 | 0.677-1.022 |  |
|  |  | Du *et al.* (2014) | 0.957 | 0.749-1.222 |  |
| rs3746444 | B vs. A | Mittal *et al.* (2011) | 1.138 | 1.034-1.251 | Fixed |
|  |  | Deng *et al.* (2015) | 1.122 | 1.023-1.231 |  |
|  |  | Hu *et al.* (2009) | 1.072 | 0.963-1.192 |  |
|  |  | Du *et al.* (2014) | 1.146 | 1.042-1.260 |  |
|  |  | Toraih *et al.* (2016) | 1.119 | 1.021-1.227 |  |
|  |  | George *et al.* (2011) | 1.119 | 1.018-1.229 |  |
|  |  | Nikolic *et al.* (2015) | 1.125 | 1.022-1.238 |  |
|  |  | Hashemi *et al.* (2016) | 1.123 | 1.023-1.234 |  |
|  |  | Ginu *et al.* (2010) | 1.161 | 1.056-1.275 |  |
|  | BA vs. AA | Mittal *et al.* (2011) | 1.212 | 0.908-1.618 | Random |
|  |  | Deng *et al.* (2015) | 1.202 | 0.905-1.597 |  |
|  |  | Hu *et al.* (2009) | 1.220 | 0.884-1.683 |  |
|  |  | Du *et al.* (2014) | 1.250 | 0.936-1.670 |  |
|  |  | Toraih *et al.* (2016) | 1.200 | 0.913-1.578 |  |
|  |  | George *et al.* (2011) | 1.126 | 0.882-1.438 |  |
|  |  | Nikolic *et al.* (2015) | 1.210 | 0.899-1.628 |  |
|  |  | Hashemi *et al.* (2016) | 1.165 | 0.888-1.528 |  |
|  |  | Ginu *et al.* (2010) | 1.335 | 1.130-1.577 |  |
|  | BA+BB vs. AA | Mittal *et al.* (2011) | 1.187 | 0.945-1.492 | Random |
|  |  | Deng *et al.* (2015) | 1.173 | 0.938-1.467 |  |
|  |  | Hu *et al.* (2009) | 1.172 | 0.913-1.505 |  |
|  |  | Du *et al.* (2014) | 1.216 | 0.972-1.521 |  |
|  |  | Toraih *et al.* (2016) | 1.167 | 0.941-1.447 |  |
|  |  | George *et al.* (2011) | 1.122 | 0.921-1.368 |  |
|  |  | Nikolic *et al.* (2015) | 1.177 | 0.932-1.487 |  |
|  |  | Hashemi *et al.* (2016) | 1.151 | 0.926-1.431 |  |
|  |  | Ginu *et al.* (2010) | 1.266 | 1.127-1.425 |  |
|  | BB vs. AA | Mittal *et al.* (2011) | 1.188 | 0.932-1.515 | Fixed |
|  |  | Deng *et al.* (2015) | 1.150 | 0.913-1.448 |  |
|  |  | Hu *et al.* (2009) | 1.008 | 0.781-1.301 |  |
|  |  | Du *et al.* (2014) | 1.166 | 0.925-1.469 |  |
|  |  | Toraih *et al.* (2016) | 1.121 | 0.889-1.413 |  |
|  |  | George *et al.* (2011) | 1.182 | 0.933-1.496 |  |
|  |  | Nikolic *et al.* (2015) | 1.159 | 0.915-1.468 |  |
|  |  | Hashemi *et al.* (2016) | 1.158 | 0.910-1.473 |  |
|  |  | Ginu *et al.* (2010) | 1.135 | 0.896-1.436 |  |
|  | BB vs. BA+AA | Mittal *et al.* (2011) | 1.041 | 0.750-1.450 | Random |
|  |  | Deng *et al.* (2015) | 1.015 | 0.739-1.396 |  |
|  |  | Hu *et al.* (2009) | 0.915 | 0.695-1.203 |  |
|  |  | Du *et al.* (2014) | 1.026 | 0.747-1.409 |  |
|  |  | Toraih *et al.* (2016) | 0.989 | 0.718-1.362 |  |
|  |  | George *et al.* (2011) | 1.108 | 0.849-1.445 |  |
|  |  | Nikolic *et al.* (2015) | 1.022 | 0.736-1.418 |  |
|  |  | Hashemi *et al.* (2016) | 1.050 | 0.759-1.452 |  |
|  |  | Ginu *et al.* (2010) | 0.938 | 0.710-1.239 |  |

B: mutated allele; A: wild allele.

**Table S3.** *P* values of the Egger’s test for the polymorphisms in miRNAs.

| **Polymorphism** | **Subgroup** | **N** | **Egger' test P>\|t\|** | **Trim and Fill Method** |
| --- | --- | --- | --- | --- |
| miR-196a2 (rs11614913) | Overall | 11 | 0.315 | - |
|  | Asian | 6 | 0.384 | - |
|  | Caucasian | 5 | 0.683 | - |
|  | H-B | 7 | **0.021** | Bias Not Existed |
|  | P-B | 4 | 0.430 | - |
|  | N | 5 | **0.008** | Bias Existed |
|  | Y | 6 | 0.539 | - |
|  | BC | 4 | 0.558 | - |
|  | PCa | 4 | 0.473 | - |
|  | RCC | 3 | 0.314 | - |
| miR-146a (rs2910164) | Overall | 11 | 0.100 | - |
|  | Asian | 9 | 0.153 | - |
|  | Caucasian | 2 | - | - |
|  | H-B | 6 | 0.074 | - |
|  | P-B | 5 | 0.938 | - |
|  | N | 4 | 0.537 | - |
|  | Y | 7 | 0.133 | - |
|  | BC | 5 | 0.437 | - |
|  | PCa | 4 | 0.798 | - |
|  | RCC | 2 | - | - |
| miR-499 (rs3746444) | Overall | 8 | 0.293 | - |
|  | H-B | 4 | 0.650 | - |
|  | P-B | 4 | 0.461 | - |
|  | N | 3 | 0.295 | - |
|  | Y | 5 | 0.317 | - |
|  | BC | 3 | 0.539 | - |
|  | PCa | 3 | 0.527 | - |
|  | RCC | 2 | - | - |

RCC: renal cell carcinoma; PCa: prostate cancer; BC: bladder cancer; H-B: hospital-based; P-B: population-based; Y: study conformed to HWE; N: study did not conform to HWE; N: number of studies.
